# Age-Related Immunoreactivity Profiles to Diverse Mycobacterial Antigens in BCG-Vaccinated Chinese Population

**DOI:** 10.3389/fimmu.2020.608220

**Published:** 2021-01-29

**Authors:** Qing-yuan Yang, Yu-tong Zhang, Jia-ni Xiao, Yu-shuo Liang, Ping Ji, Shu-jun Wang, Ying Wang, Yingying Chen

**Affiliations:** ^1^ Department of Microbiology and Immunology, Shanghai Institute of Immunology, Shanghai Jiao Tong Unviersity School of Medicine, Shanghai, China; ^2^ Key Laboratory of Parasite and Vector Biology, Ministry of Health, School of Global Health, Chinese Center for Tropical Diseases Research, Shanghai Jiao Tong University School of Medicine, Shanghai, China

**Keywords:** BCG vaccination, mycobacterial proteins, immunoreactive profiles, healthy population, aging

## Abstract

Long-term immunoreactivity to mycobacterial antigens in Bovis Calmette-Guérin (BCG)-vaccinated population is not well investigated. Herein, 361 volunteer healthy donors (HDs) with neonatal BCG vaccination from Shanghai region (China) were enrolled. They were subdivided into ESAT-6/CFP10^-^ (E6C10^-^) and ESAT-6/CFP10^+^ (E6C10^+^) groups based on gamma-interferon release assays (IGRAs). Three mycobacterial antigens, including Rv0934, Rv3006, and Rv3841, were subjected to the determination of immunoreactivity by ELISPOT assay. The immunoreactivities to three mycobacterial antigens were firstly compared among TB patients (N=39), E6C10^+^ HDs (N=78, 21.61% of HDs) and E6C10^-^ HDs (N=283, 78.39% of HDs). It was revealed that Rv3006 was dominant upon *M.tb* infection, while Rv3841 was likely to be more responsive upon latent TB infection. In E6C10^-^ population, the immunoreactivity to Rv3841 maintained along with aging, whereas those to Rv3006 and Rv0934 attenuated in E6C10^-^ HDs older than 45 years old. Our study implies the shift of dominant antigens at different infection statuses, providing the clues for the selection of mycobacterial antigens in vaccine development and precision revaccination in the future.

## Introduction

Tuberculosis (TB) is an infectious disease caused by *Mycobacterium tuberculosis* (*M.tb*). It is the leading cause of the death by a single infection. Ten million people are estimated to develop TB in 2018 globally (1). Bovis Calmette-Guérin (BCG) is the live vaccine derived from attenuated *Mycobacterium bovis* and was firstly used in 1921. It is currently the only available TB vaccine recommended by the World Health Organization (WHO) to be vaccinated in the countries with high burden of TB. China has implemented BCG planned immunization for all newborns since 1957 (2). However, it is estimated that the protection of BCG lasts for merely 15~20 years (3, 4), which is derived from the epidemiological observation that there occurs the increased incidence of TB in teenagers (5). More seriously, 10% of *M.tb*-infected population will develop into latent tuberculosis infection (LTBI), in which *M.tb* persistently exists in the hosts with no clinical manifestations. Therefore, developing new vaccines to prevent *M.tb* infection and the reactivation of LTBI in adults become one of the key challenges for TB control.

BCG vaccination in newborns can trigger efficient innate and adaptive immunity. After vaccination, live BCG can be captured by macrophages or dendritic cells. Mycobacterial antigens are presented by MHC molecules to induce type 1 helper T cell (Th1) immune responses including the activation of antigen-specific CD4^+^ and CD8^+^ T cells (6). Memory immune responses to BCG are generated thereafter to exert immune protection in the long run. Neonatal BCG vaccination can dramatically decrease the mortality of both *M.tb* infection and other pathogen infections (7). In adults, BCG can decrease the incidence of allergic diseases such as asthma (8), and enhance antibody responses induced by influenza vaccine (9). The difference in the resistance to *M.tb* infection in adults, to some extent, is probably related to memory responses generated through neonatal BCG vaccination (10).

BCG contains more than 4,000 proteins, among which some are immunodominant once vaccinated in the hosts. However, whether the dominant antigen profiles shift along with the age is not well investigated. In this study, three *M.tb* and BCG-shared mycobacterial antigens, Rv3006, Rv3841 and Rv0934, were subjected to the investigation due to their different immunogenicity and immunoreactivity upon *M.tb* infection based on our previous studies (11, 12). Rv3006 (also known as lipoprotein Z, LppZ) is both a cell-wall component and a secreting protein (13). Studies from our own and other groups demonstrate that it is of high immunogenicity during *M.tb* infection triggering both innate and adaptive immunity (11, 12, 14, 15). Rv3841 (also known as BfrB, a ferritin-like protein) is reported to be an iron-storage protein (16). It is involved in the survival and pathogenesis of *M.tb* in the hosts. Rv3841-specific cellular response is much lower in active pulmonary TB patients when compared to early secreted antigenic target 6 (ESAT-6) and culture filtrate protein 10 (CFP10), two well-known dominant *M.tb*-specific antigens, whereas comparable to ESAT-6 and CFP10 in LTBI individuals. More interestingly, there exist a certain percentage of healthy donors (HDs) with high Rv3841-specific cellular responses, which is probably cautionary in identifying leaky LTBI from HDs (17). Rv0934 (also known as PstS1/Phos1) belongs to the family of ATP-binding cassette transporters. It is absent in most of non-tuberculous mycobacteria (NTM) except *Mycobacterium malmoense*, *Mycobacterium intracellulare, etc*. Rv0934 occurs in fewer amounts in the BCG strain (18, 19) and is likely a humoral immunodominant protein (20). Rv0934-specific humoral response is considered as a potential biomarker for the diagnosis of active TB (21–23). Quantitative detection of Rv0934 might monitor the progression of TB (24).

Considering the diverse immunoreactivity patterns of three mycobacterial antigens upon *M.tb* infection, herein we aimed to characterize the cellular immune response patterns of Rv0934, Rv3006, and Rv3841 in BCG-vaccinated healthy cohorts and TB patients in different age groups. Accordingly, antigen-specific response profiles induced by neonatal BCG vaccination were introduced, which might be helpful to define mycobacterial proteins suitable for vaccine development in BCG-vaccinated population.

## Methods and Materials

### Study Subjects

A total of 361 healthy volunteers were included in this study. They underwent annual physical examination in Ruijin Hospital affiliated to Shanghai Jiao Tong University School of Medicine, including blood tests, serum biochemical indexes, chest X-rays with no medical history or disease symptoms. They were vaccinated with the BCG Shanghai strain (Shanghai Institute of Biological Products Co., Ltd., Shanghai, China) at newborn. Gamma-interferon (IFN-γ) releasing assays (IGRAs) using synthesized ESAT-6 (E6p) or CFP10 (C10p) peptide pools were performed by an enzyme-linked immunospot (ELISPOT) assay to distinguish ESAT-6/CFP10^-^ (E6C10^-^) and ESAT-6/CFP10^+^ (E6C10^+^) healthy donors (HDs). E6C10^+^ HDs (N=78) were defined as those with more than 6 spot forming units (SFUs)/2.5×10^5^ PBMCs either specific to E6p or C10p. E6C10^-^ individuals (N=283) were defined as those with SFUs < 6. TB patients (N=39) were in-patient patients from Shanghai Public Health Clinical Center (Shanghai, China). TB was confirmed based on medical history, chest radiograph (X-ray and CT), acid-fast bacilli (AFB) smear or sputum culture. They were both HIV- and HBV-negative. All TB patients had signed voluntary informed consents before being enrolled. This study was approved by the Ethics Committee of Shanghai Jiao Tong University School of Medicine.

### Preparations of Recombinant Mycobacterial Antigens

Recombinant plasmids were obtained from NIH Biodefense and Emerging Infection Research Resources Repository (NIAID, NIH, USA), including pMRLB.5 containing *rv3841* (Protein BfrB) (NR-13278), pMRLB.2A containing *rv0934* (Protein PstS1/PhoS1) (NR-13277) and pMRLB.54 containing *rv3006* (Protein LppZ) (NR-13304). Recombinant mycobacterial proteins were expressed prokaryotically and purified by affinity chromatography with Ni-NTA His-Bind Resin (Qiagen, NRW, Germany) accordingly (11, 17). Endotoxins were removed from purified antigens using Triton X-114 (Sigma-Aldrich, Missouri, USA) two-phase separation (11). According to the manufacturer’s protocol, the remaining endotoxin in the proteins was detected by Tachyleus Amebocyte Lysate Kits (Gulangyu, Xiamen, China). Protein concentration was quantified by BCA Protein Assay Kit (Pierce, Waltham, MA, USA).

### Determination of the Purity of Recombinant Proteins

The purity of the proteins was determined by Coomassie brilliant blue staining and further confirmed by Western blotting. Three recombinant proteins were separated by 12% sodium dodecyl sulfate-polyacrylamide gel electrophoresis (SDS-PAGE) (Bio-Rad Laboratories Inc., USA), and were electro-transferred to polyvinylidene fluoride (PVDF) membrane (Millipore, MA, USA) after electrophoresis. An anti-6×His tag antibody (Abcam, Hong Kong, China) (11) was used as primary antibody and horseradish peroxidase (HRP)—conjugated goat anti-mouse IgG (Cwbio, Beijing, China) was used as secondary antibody. The ECL solution (Millipore) was used to visualize Rv3006, Rv3841, and Rv0934 proteins. The membranes were scanned on Tanon 5200S Chemiluminescence Imaging System (Tanon, Shanghai, China).

### Preparation of Peripheral Blood Mononuclear Cells

Ten microliters of whole blood was collected in tubes containing EDTA. Peripheral blood mononuclear cells (PBMCs) were isolated by Ficoll-hypaque density gradient centrifugation with Lymphoprep™ solution (AXIS-SHIELD Poc AS, Oslo, Norway) at 860 *g* for 20 min at room temperature (RT) (17). The mononuclear cells were carefully transferred and washed with RPMI 1640 (GIBCO, Grand Island, USA). PBMCs were resuspended at a concentration of 2.5×10^6^/ml in RPMI 1640 culture medium containing 10% fetal bovine serum (FBS) (Merck Millipore, Darmstadt, Germany), 100 units/ml penicillin and 100 µg/ml streptomycin (both from GIBCO).

### Gamma-Interferon Release Assays (IGRAs)

Antigen-specific IFN-γ responses were detected by an ELISPOT assay (U-CyTech Biosciences) according to the manufacturer’s instructions (17). Briefly, 96-well polyvinylidene fluoride (PVDF) plates (Millipore) were coated with anti-human IFN-γ coating antibody overnight at 4°C. 2.5 × 10^5^ PBMCs in 100 µl RPMI 1640 culture medium was plated in each well and stimulated with E6p or C10p peptide pools (2 µg/ml per peptide, Sangon Biotech, Shanghai, China), or purified Rv0934, Rv3006, and Rv3841 proteins (2 µg/ml). Culture medium served as a negative control, and 2 µg/ml phytohemagglutinin (PHA) (Sigma-Aldrich, St. Louis, MO, USA) was used as a positive control. After 20 h incubation at 37°C, the plates were incubated with biotin-labeled detection antibody at 37°C for 1 h and horseradish peroxidase (HRP)-conjugated streptavidin in working solution for another 1 h. AEC substrate solution was added to each well for less than 30 min in the dark at RT. Color development was stopped by thoroughly rinsing both sides of the PVDF membrane with demineralized water. The plates were dried in the dark at RT. The spots were counted by ELISPOT BioReader (CTL S6 Ultra, Ohio, USA). The number of antigen-specific IFN-γ-producing cells was calculated based on SFUs per 2.5 × 10^5^ PBMCs after deducting the background SFUs in paired negative control wells. Individuals that did not meet the criteria of positive (SFU > 5) or negative (SFU < 20) controls were ruled out.

### Statistical Analysis

Data was represented by median with interquartile range (median, 25% Percentile-75% Percentile). Statistical analyses were performed by GraphPad Prism 7.0 software (GraphPad Software Inc., CA, USA). All the data are non-normal distribution and nonparametric analysis is applied in the study. Statistical differences were then assessed by the Kruskal-Wallis test and by Dunn’s multiple comparison test accordingly. Correlations were analyzed by Spearman’s nonparametric correlation analysis. The *p*-values < 0.05 were considered statistically significant.

## Results

### Study Subjects

361 volunteer healthy donors (HDs) were enrolled in this study. There was no difference in the gender between HDs (female/male: 167/194) and TB patients (female/male: 21/18), while TB patients (median age: 52 years old; interquartile range: 35–65) were older than HDs (median age: 39 years old; interquartile range: 29–50) (*p* < 0.0001) (**Table 1**). By using E6p and C10p-specific IGRAs, 78 subjects were screened out as E6C10^+^ (median age: 38 years old; interquartile range: 27–51; female/male: 34/44) and 283 individuals were E6C10^-^ (median age: 39 years old; interquartile range: 29–50; female/male: 133/150). The percentage of E6C10^+^ healthy donors was 21.61%, which is close to the estimation of 23% as reported by the WHO (1). However, no strong correlations were observed between EAST-6- or CFP10-specific IFN-γ releasing cells and the ages in E6C10^+^ HDs (**Figure S1**).

**Table 1:** Basic information of subjects.

	Healthy Donors			TB
	Total	E6C10^-a^	E6C10^+b^	
N	361	283	78	39
Gender (F/M)	167/194	133/150	34/44	21/18
Age (years old)				
median	39	39	38	52
interquartile range	29–50	29–50	27–51	35–65

N; sample size, F; female, M; male.

^a^ESAT-6/CFP10^-^; ^b^ESAT-6/CFP10^+^.

### Distinct Immunoreactivity to Rv0934, Rv3006, and Rv3841 in E6C10^-^ and E6C10^+^HD Populations and TB Patients

**Figure 1:**
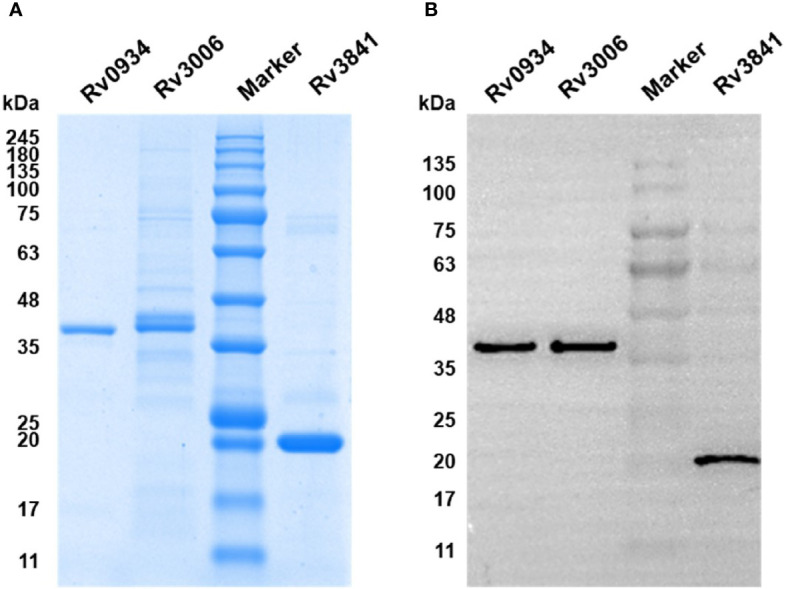
Expression and identification of recombinant proteins Rv0934, Rv3006, and Rv3841. **(A)** Identification of the purity of prokaryotically expressed Rv0934, Rv3006, and Rv3841 recombinant proteins by sodium dodecyl sulfate-polyacrylamide gel electrophoresis (SDS-PAGE), stained with Coomassie brilliant blue. **(B)** Verification of purified Rv0934, Rv3006, and Rv3841 proteins by western blotting. Purified His-tagged proteins were confirmed with mouse anti-6×His tag antibody and HRP conjugated goat anti-mouse IgG antibody. Molecular mass: Rv0934 Protein: 38 kDa; Rv3006 Protein:38 kDa; Rv3841 Protein: 20 kDa.

To explore the antigen-specific immunoreactive profiles induced by neonatal BCG vaccination, three mycobacterial proteins shared by *M.tb* and BCG, including Rv0934, Rv3006, and Rv3841, were selected based on our previous studies. Three mycobacterial proteins were inductively expressed in *E. coli* BL21 (DE3) and purified by Ni-NTA His-Bind Resin. The purity was identified by using SDS-PAGE with the expected molecular weight of 38 kDa (Rv0934 and Rv3006) and 20 kDa (Rv3841) (**Figure 1A**), respectively, and were further confirmed by Western blotting with an anti-6×His tag antibody (**Figure 1B**).

**Figure 2:**
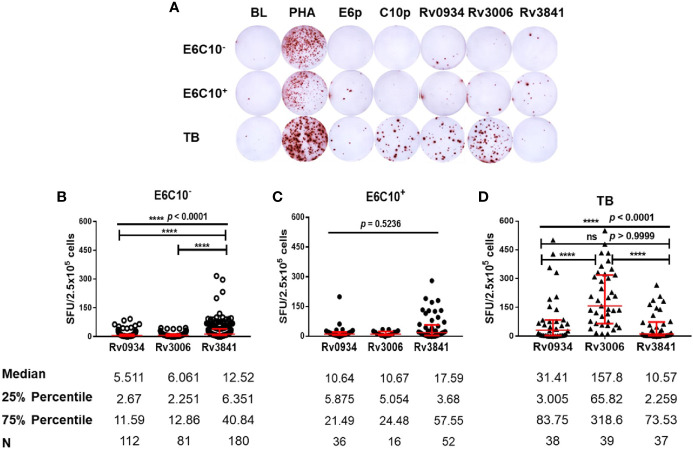
Different gamma-interferon (IFN-γ) response patterns targeting Rv0934, Rv3006 and Rv3841. **(A)** Representative results of enzyme-linked immunospot (ELISPOT) assay. Peripheral blood mononuclear cells (PBMCs) were stimulated with different antigens for 20 h, including *M.tb*-specific ESAT-6 peptide (E6p) and CFP10 peptide (C10p) as well as three mycobacterial recombinant proteins. Culture medium served as the negative control (BL), while phytohemagglutinin (PHA) as the positive control. **(B–D)** Comparison of IFN-γ responses of Rv0934, Rv3006, and Rv3841 in E6C10^-^ population **(B)**, E6C10^+^ population **(C)**, and in tuberculosis (TB) patients **(D)**. The *p*-values were calculated using the Kruskal-Wallis test and Dunn’s multiple comparison test. The *p*-values above the lines are the *p*-values of the Kruskal-Wallis test. The *p*-values above capped lines are the adjusted *p*-value of Dunn’s multiple comparison test. ****p < 0.0001.

Freshly isolated PBMCs from HDs and TB patients were stimulated *in vitro* by synthesized peptide pools (E6p or C10p) as well as recombinant mycobacterial proteins (Rv0934, Rv3006, and Rv3841) (**Figure 2A**). Based on IGRA assays specific to E6p and C10p, HDs were subdivided into E6C10^-^ and E6C10^+^ groups according to the SFU values with the cutoff of 6 per 2.5 × 10^5^ PBMCs. Firstly, we compared IFN-γ responses upon stimulation with Rv0934, Rv3006, and Rv3841 in E6C10^-^ HDs, E6C10^+^ HDs and TB patients, respectively (**Figures 2B–D**). In E6C10^-^ population, the level of Rv3841-specific IFN-γ producing cells was the highest (median SFUs/2.5 × 10^5^ PBMCs: 12.52; interquartile range: 6.351–40.84) whereas those of Rv0934 (median SFUs/2.5 × 10^5^ PBMCs: 5.511; interquartile range: 2.67–11.59) and Rv3006 (median SFUs/2.5 × 10^5^ PBMCs: 6.061; interquartile range: 2.251–12.86) were relatively low (*p* < 0.0001) (**Figure 2B**). Meanwhile, the immunoreactivity to Rv3841 was also the highest with a median value of 17.59 SFUs/2.5 × 10^5^ PBMCs (interquartile range: 3.68–57.55) in E6C10^+^ population (*p* = 0.5263) (**Figure 2C**). Our results also revealed that Rv3006 induced the strongest immunoreactivity in TB patients with a median value of 171.6 SFUs/2.5 × 10^5^ PBMCs (interquartile range: 67.94–322.5) when compared to Rv0934 (median SFUs/2.5 × 10^5^ PBMCs: 27.37; interquartile range: 3.125–83.48) and Rv3841 (median SFUs/2.5 × 10^5^ PBMCs: 9.424; interquartile range: 2.424–72.2) (*p* < 0.0001) (**Figure 2D**). The levels of Rv0934 and Rv3006-specific IFN-γ producing cells were significantly higher in TB than those in HDs (for Rv0934 *p* = 0.0010; for Rv3006: *p* < 0.0001) (**Figures S2A, B**). However, the immunoreactivity to Rv3841 was slightly lower in TB patients than those in E6C10^-^ and E6C10^+^ HD (*p* = 0.8238) (**Figure S2C**).

### Rv3006 and Rv0934 Exhibit Attenuated Performance in E6C10^-^ Elderly Population While Rv3841 Maintains the Immunoreactivity Along With Aging

**Figure 3:**
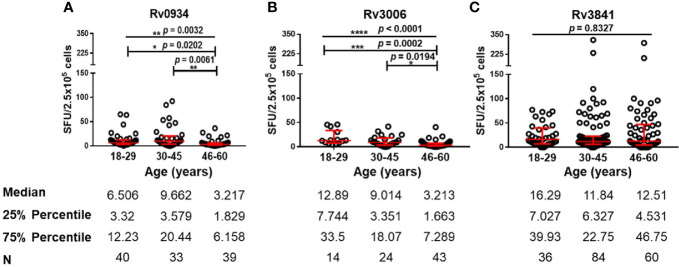
Numbers of mycobacterial antigen-specific gamma-interferon (IFN-γ) releasing cells in E6C10^-^ population. **(A–C)** Rv0934 **(A)**-, Rv3006 **(B)**-, and Rv3841 **(C)**- specific IFN-γ releasing cell numbers in peripheral blood mononuclear cells (PBMCs) from different age groups of E6C10^-^ subjects. The *p*-values were calculated using the Kruskal-Wallis test and Dunn’s multiple comparison test. The *p*-values above the lines are the *p*-values of the Kruskal-Wallis test. The *p*-values above capped lines are the adjusted *p*-value of Dunn’s multiple comparison test. ****p < 0.0001. ***p < 0.001, **p < 0.01. *p < 0.05.

Since all the HDs received BCG vaccination according to the national planned vaccination program, we further analyzed the immunoreactivity to three antigens at different ages. Both E6C10^-^ and E6C10^+^ HDs were further subgrouped into young (18–29 years), middle-aged (30–45 years), and elderly groups (46–60 years). The immunoreactivity levels to three proteins at different ages were compared in E6C10^-^ HD population. In the group of elderly individuals, a dramatic decline of antigen-specific IFN-γ producing cells was observed when compared to the younger groups upon *in vitro* stimulation of Rv0934 (18–29 years *vs.* 46–60 years: *p* = 0.0202; 30–45 years *vs.* 46–60 years: *p* = 0.0061) (**Figure 3A**) and Rv3006 (18–29 years *vs.* 46-60 years: *p* = 0.0002; 30–45 years *vs.* 46–60 years: *p* = 0.0194) (**Figure 3B**). The number of Rv0934-specific IFN-γ-releasing cells was weakly correlated with age (Rv0934: r = −0.1976, *p* = 0.0367) (**Figure S3A**). Interestingly, the number of Rv3006-specific IFN-γ-releasing cells gradually decreased with the increase in the ages (Rv3006: r = −0.4745, *p* < 0.0001) (**Figure S3B**). However, the number of Rv3841-specific IFN-γ releasing cells did not fluctuate along with aging (Rv3841: r = −0.07624, *p* = 0.3090) (**Figure S3C**) and among the three age groups (*p* = 0.8327) (**Figure 3C**).

### The Immunoreactivity Profiles to Rv0934, Rv3006, and Rv3841 in E6C10^+^ HD Population

**Figure 4:**
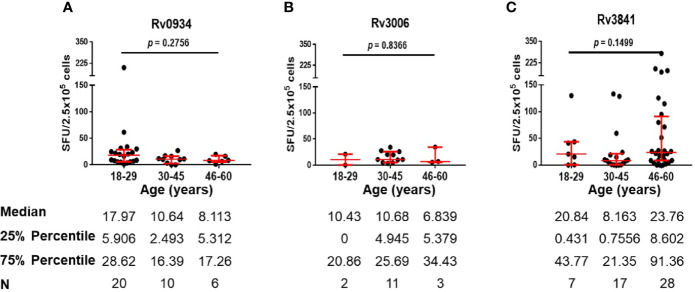
Numbers of mycobacterial antigen-specific gamma-interferon (IFN-γ) releasing cells in E6C10^+^ population. **(A–C)** Rv0934 **(A)**-, Rv3006 **(B)**-, and Rv3841 **(C)**- specific IFN-γ releasing cell numbers in peripheral blood mononuclear cells (PBMCs) from different age groups of E6C10^+^ subjects. The *p*-values were calculated using the Kruskal-Wallis test and Dunn’s multiple comparison test.

E6C10^+^ HDs are defined as SFUs value more than six by IGRA assays specific to ESAT-6 and CFP10, two dominant *M.tb*-specific antigens, and is thought to be infected previously by *M.tb* or latent TB infection individuals. Antigen-specific immune responses are supposed to be induced by transient *M.tb* infection combined with neonatal BCG vaccination. We compared the numbers of IFN-γ-releasing cells induced by three antigens in E6C10^+^ HDs at different age groups. Rv0934 displayed the strongest immunoreactivity at the younger period (18–29 years) while decreased afterward (**Figure 4A**). Although Rv3006 was the most immunodominant among three antigens in TB patients (**Figure 2D**), there was no statistical difference in Rv3006-triggered IFN-γ releasing cells among age groups with low level of antigen-specific immunoreactivity in E6C10^+^ HDs (**Figure 4B**). On the other hand, the numbers of Rv3841-specific IFN-γ-releasing cells were more in the 18-29- and 46- to 60-year-old groups than that in the 30- to 45-year-old group in E6C10^+^ HDs (**Figure 4C**). Besides, antigen-specific IFN-γ-releasing cells had no correlation with the ages in E6C10^+^ HD population (Rv0934: r = −0.1843, *p* = 0.2820; Rv3006: r = 0.06647, *p* = 0.8064; Rv3841: r = 0.07699, *p* = 0.5875) (**Figures S3D–F**). These data demonstrate that Rv3841 remains the high immunoreactivity along with aging, while Rv3006 and Rv0934 are not so immunoreactive upon latent *M.tb* infection.

### The Immunoreactivity Profiles to Rv0934, Rv3006, and Rv3841 in TB Patients

**Figure 5:**
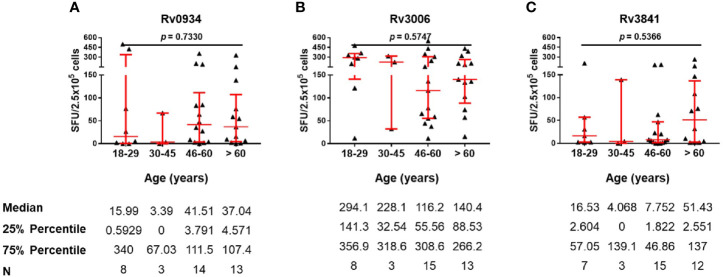
Numbers of mycobacterial antigen-specific gamma-interferon (IFN-γ) releasing cells in TB patients. **(A–C)** Rv0934 **(A)**-, Rv3006 **(B)**-, and Rv3841 **(C)**- specific IFN-γ releasing cell numbers in peripheral blood mononuclear cells (PBMCs) from different age groups of tuberculosis (TB) patients. The *p*-values were calculated using the Kruskal-Wallis test.

TB patients were those with acute infection of *M.tb*. Three antigens shared between BCG and *M.tb* were able to induce immunoreactivity in TB patients as well (**Figure 2D**). We therefore compared antigen-specific immunoreactivities in TB patients along with aging. In general, there were no significant differences in the levels of antigen-specific IFN-γ releasing cells among the different age groups (**Figure 5**). In more detail, Rv0934 induced relatively stronger responses in TB patients over 46 years old (**Figure 5A**), whereas the immunoreactivity to Rv3006 maintained along the ages (**Figure 5B**). A similar tendency was observed in the Rv3841-specific cellular response, which exhibited a larger number of IFN-γ releasing cells in the eldest age group (**Figure 5C**).

## Discussion

Neonatal BCG vaccination in TB high burden countries including China is demonstrated to induce innate and adaptive immunity. At 3 months after neonatal BCG vaccination, 62.0% of the infants had purified protein derivative (PPD) readings of 5-10 mm induration size, and 35.2% had readings of larger than 10-15 mm size (25). The immune profiles during the early stages of BCG vaccination have also been investigated. BCG engages in the innate immunity mainly *via* pathogen recognition receptors (PRRs), including Toll-like receptor (TLR)-2, -4, -7, -8, and -9 as well as C-type lectin receptors and induces cytokine responses (26). In the periphery, cytokines including IL-1, IL-6, and MIP-1α have been elevated in BCG vaccinated infants at 3 months when compared to those without vaccination (26). BCG-specific CD4^+^ T cell responses peak 6-10 weeks after neonatal vaccination and gradually ebb within the first year in which Th1 cells play a significant role. CD45RA^-^CCR7^+^ central memory T cells become predominant to conduct protective effects in the following period (27). Another report described the immune profiles in the children within 5 years old (28). It was found that PPD-specific responses sustained 5 years after neonatal BCG vaccination, which was demonstrated to guarantee the protection against TB in childhood. However, the increased incidence of TB from 15 years old (5) implies the attenuation of BCG vaccination along with aging. Therefore, new vaccines are required to decrease the incidence of TB. However, how to choose mycobacterial antigens for vaccine development is ambiguous. In this study we were inclined to determine the immunoreactivity to three mycobacterial antigens in the adults, and to evaluate their immunodominance and immune sustainability in the long term after neonatal BCG vaccination.

Different immunological patterns were revealed under either *M.tb* infection or neonatal BCG vaccination among E6C10^-^, E6C10^+^ HDs and TB cohorts. Consistent with previous studies (11, 12, 14, 15), Rv3006 is of high immunogenicity upon *M.tb* infection, with the most IFN-*γ* specific SFUs in TB patients (**Figure S1B**). However, BCG vaccination does not induce strong immunoreactivity to Rv3006 in E6C10^-^ and E6C10^+^ adults (**Figure S1B**). The reason might lie in the fact that TB patients are infected by a high dose of *M.tb* bacilli through the respiratory tract. The immune responses can be triggered rapidly by cell wall components or secreting proteins (like Rv3006) exhibiting immune dominance. BCG is injected through an intra-muscular pathway with less amount of Rv3006 release, making the capture of Rv3006 by antigen presenting cells (APCs) with a low dosage and subsequently less immunogenicity. This is consistent with the observation that Rv3006 exhibits low immunoreactivity under LTBI, which is supposed to be of low burden of bacilli infection.

On the contrary, the immunoreactivity to Rv3841 in TB patients is low when compared to Rv3006 and Rv0934 (**Figure 2D**). Rv3841 is more likely to be dominant upon BCG vaccination (**Figure S2C**). As an intracellular antigen, Rv3841 cannot be accessible as quickly as membrane or secretory antigens by APCs to induce an immediate immune response upon *M.tb* infection. However, BCG vaccination is more inclined to make macrophages or dendritic cells engulfing whole bacilli to trigger systematic immune responses. Rv3841 is likely to be more dominant in this situation. It is also surprising that Rv3841 triggers a stronger immune response under LTBI status (E6C10^+^ HDs) (**Figure S2C**), representing the persistence of bacilli with less active status in the macrophages. Sustained immunoreactivity to Rv3841 in E6C10^-^ HDs along with aging (**Figure 3C**) suggests that Rv3841-induced IFN-γ response is more long-lasting than Rv3006 and Rv0934.

The distinct response patterns induced by three proteins may be also attributed to their expression properties. Rv0934 and Rv3006 are secretory antigens while Rv3841 is an intracellular antigen. Besides, Ottenhoff and Kaufmann (29) reported that antigen-induced protective efficacy and T cell responses were closely relevant to the antigen dose. Excessive antigen dose reduces the longevity of protection against TB in the mice (30). Hence, the protein levels in BCG might be one of the factors to explain the discrepancies in immune response profiles of the three antigens.

Moreover, the diverse immune response profiles could be, in part, determined by the disease state. In TB patients in acute *M.tb* infection status, no significant differences in the antigen-specific response along with aging were observed (**Figure 5**). The immune responses of the three mycobacterial antigens were further compared in both E6C10^-^ and E6C10^+^ healthy subpopulations. These two populations are both potential revaccination targets. However, they represent different infection statuses. While E6C10^-^ subpopulations are those only with neonatal BCG vaccination, E6C10^+^ subpopulations are more likely to be latently or previously infected by *M.tb* with a high incidence of reactivation (1, 5). Unexpectedly, the immune response to Rv3006 is still lower than Rv3841 in healthy population. This might be associated with a low dose of *M.tb* bacilli infection, which is not strong enough for Rv3006 to induce antigen-specific immune responses. However, the immune responses to Rv3841 are more apparent in E6C10^+^ subpopulations than E6C10^-^ counterpart (**Figure S2C**), which implies that Rv3841 might be superior to Rv3006 as a boosting antigen candidate for E6C10^+^ subpopulations. On the other hand, considering the high immunogenicity of Rv3006 upon *M.tb* infection and low responsiveness in the healthy population (in both E6C10^-^ and E6C10^+^ subpopulations) together with the evidence on its immunoprotection in immunized mice, Rv3006 vaccination might exert good protection once upon *M.tb* infection. These deductions need to be validated in the future in BCG priming-boosting animal models.

## Conclusion

In conclusion, our results presented here elucidate that immunoreactivity patterns of mycobacterial antigens upon BCG vaccination are different from those upon *M.tb* infection. Since ESAT-6/CFP10^-^ HDs represents those with non-infection of *M.tb* bacilli in BCG vaccinated population, diverse immune patterns of mycobacterial antigens along aging imply the existence of immunoreactivity segregation after BCG vaccination, which might provide important clues for the selection of mycobacterial antigens for vaccine development.

## Data Availability Statement

The raw data supporting the conclusions of this article will be made available by the authors, without undue reservation.

## Ethics Statement

The studies involving human participants were reviewed and approved by the Ethical Committee of Shanghai Jiao Tong University School of Medicine. The ethics committee waived the requirement of written informed consent for participation.

## Author Contributions

Conceived the project and designed experiments: YC and YW. Performed the experiments: QY, YZ, JX, and YL. Collected samples: PJ, SW, and YC. Analyzed data: QY, YZ, YC, and YW. Wrote the manuscript: QY, YZ, and YW. Obtained funding: YC and YW. All authors contributed to the article and approved the submitted version.

## Funding

This work was supported by grants from Chinese National Mega Science and Technology Program on Infectious Diseases (2018ZX10731301-001-004, 2018ZX10302301-002-002, and 2013ZX10003007-003-003), Shanghai Academic Research Leader Project (18XD1403300), National Natural Science Foundation of China (81873868, 81501361), and Science and Technology Commission of Shanghai Municipality (19ZR1445800).

## Conflict of Interest

The authors declare that the research was conducted in the absence of any commercial or financial relationships that could be construed as a potential conflict of interest.
