# Supplementary material for: Age-Related Immunoreactivity Profiles to Diverse Mycobacterial Antigens in BCG-Vaccinated Chinese Population
Source: Front Immunol. 2021 Jan 29;11:608220. doi: 10.3389/fimmu.2020.608220 (PMC7878369; doi:10.3389/fimmu.2020.608220)
Supplement: Supplementary file 1 [file DataSheet_1.docx]

Supplementary Material

Age-related immunoreactivity profiles to diverse mycobacterial antigens in BCG vaccinated Chinese population

Qing-yuan Yang^1,#^, Yu-tong Zhang^1,#^, Jia-ni Xiao^1^, Yu-shuo Liang^1^, Ping Ji^1^, Shu-jun Wang^1^, Ying Wang^1,2,*^, Yingying Chen^1,*^

1 Department of Microbiology and Immunology, Shanghai Institute of Immunology, Shanghai Jiao Tong University School of Medicine, Shanghai, China

2 Key Laboratory of Parasite and Vector Biology, Ministry of Health, School of Global Health, Chinese Center for Tropical Diseases Research, Shanghai Jiao Tong University School of Medicine, Shanghai, China

# Supplementary Figures


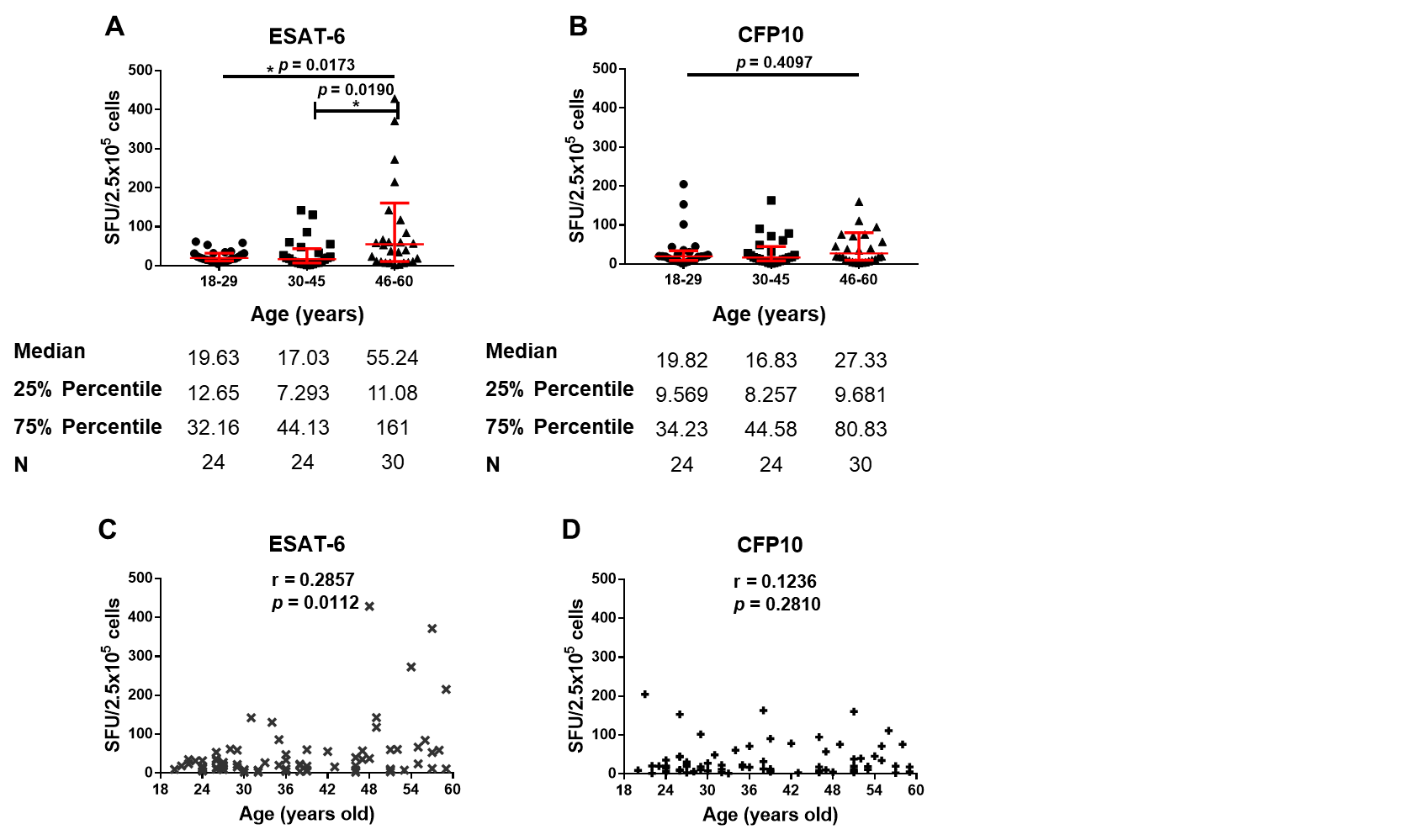


**Supplementary Figure 1** Correlations between ESAT-6/CFP10 assay and the ages in E6C10^+^ HD subjects. (A-B) Comparisons of ESAT-6 (A)- and CFP10 (B)-specific IFN-γ releasing cells in different age groups. The *p*-values were calculated using the Kruskal-Wallis test and Dunn’s multiple comparison test. The *p*-values above the lines were the *p*-values of the Kruskal-Wallis test. The *p*-values above capped lines were the adjusted *p*-values of Dunn’s multiple comparison test. *: *p*＜0.05. (C-D) Correlation analyses between the ages and ESAT-6 (C)- and CFP10 (D)-specific IFN-γ releasing cell numbers. Correlations were tested by Spearman’s nonparametric correlation analysis.


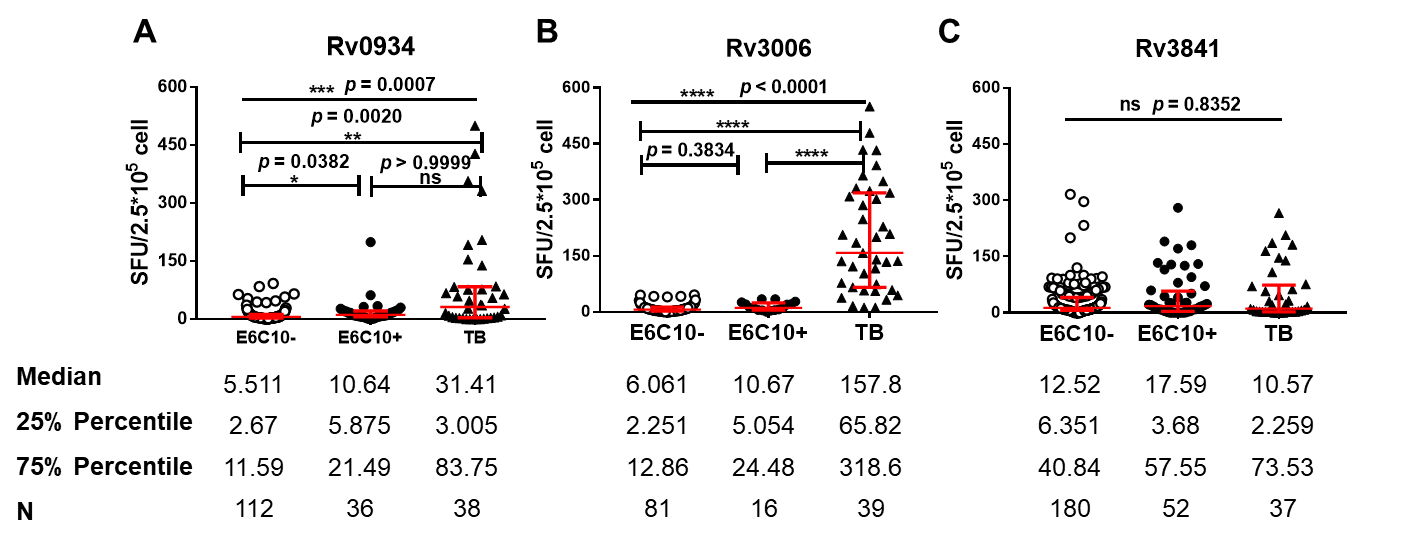


**Supplementary Figure 2** Immunoreactivities to three mycobacterial antigens in E6C10^-^ HDs, E6C10^+^ HDs and TB subjects. Numbers of Rv0934 (A)-, Rv3006 (B)-, and Rv3841 (C)- specific IFN-γ releasing cells were compared among E6C10^-^ HDs (open circle), E6C10^+^ HDs (closed circle), and TB patients (closed triangle), respectively. The *p*-values were calculated using the Kruskal-Wallis test and Dunn’s multiple comparison test. The *p*-values above the lines were the *p*-values of the Kruskal-Wallis test. The *p*-values above capped lines were the adjusted *p*-values of Dunn’s multiple comparison test. ****: *p*＜0.0001, ***: *p*＜0.001, **: *p*＜0.01, *: *p*＜0.05.

**
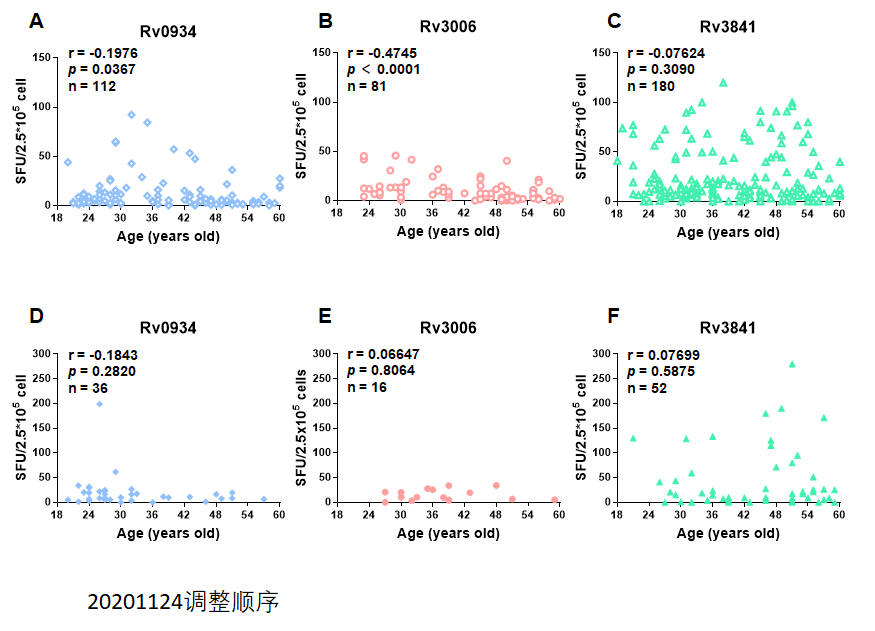
**

**Supplementary Figure 3** Correlation analyses between antigen-specific immune response levels and the ages. **(A-C)** Correlation analyses between the numbers of Rv0934 (A)-, Rv3006 (B)-, Rv3841 (C)- specific IFN-γ releasing cells and the ages in E6C10^-^ HDs. **(D-F)** Correlation analyses between the numbers of Rv0934 (D)-, Rv3006 (E)-, Rv3841 (F)- specific IFN-γ releasing cells and the ages in E6C10^+^ subjects. Correlation was tested by Spearman’s non-parametric correlation analysis.
